# Vitamin D Receptor Polymorphism and DHCR7 Contribute to the Abnormal Interplay Between Vitamin D and Lipid Profile in Rheumatoid Arthritis

**DOI:** 10.1038/s41598-019-38756-8

**Published:** 2019-02-22

**Authors:** Javier Rodríguez-Carrio, Mercedes Alperi-López, Manuel Naves-Díaz, Adriana Dusso, Patricia López, Francisco Javier Ballina-García, Jorge B. Cannata-Andía, Ana Suárez

**Affiliations:** 1 Area of Immunology, Department of Functional Biology, University of Oviedo, Oviedo, Spain; 2 Bone and Mineral Research Unit, REDinREN del ISCIII, Hospital Universitario Central de Asturias, Oviedo, Spain; 3 Instituto de Investigación Sanitaria del Principado de Asturias (ISPA), Oviedo, Spain; 4 Department of Rheumatology, Hospital Universitario Central de Asturias, Oviedo, Spain

## Abstract

Emerging evidence suggests a role for 7-dehydrocholesterol reductase (DHCR7) in the crosstalk between cholesterol and vitamin D. Our aim was to evaluate the impact of vitamin D-related polymorphisms and DHCR7 levels in the association between vitamin D deficiency and altered lipid profile in rheumatoid arthritis (RA). Serum 25(OH)-vitamin D, DHCR7 levels and vitamin D-related polymorphisms (VDR-rs2228570, CYP27A1-rs933994, CYP2R1-rs10741657 and DHCR7-rs12785878) were analyzed in 211 RA patients,94 controls and in a prospective cohort of 13 RA patients undergoing TNFα-blockade. Vitamin D was decreased in RA (p < 0.001), correlated to HDL-cholesterol (r = 0.217, p < 0.001) and total-/HDL-cholesterol ratio (r = −0.227, p = 0.004). These correlations were restricted to the VDR-rs2228570 status. Vitamin D deficiency was associated with lower HDL-cholesterol (p = 0.028), higher tender (p = 0.005) and swollen (p = 0.002) joint counts, higher DAS28 (p = 0.018) and HAQ (p = 0.024) in AG/AA-patients but not in their GG-counterparts. The associations among DHCR7, vitamin D and lipid profile followed a seasonal pattern, decreased DHCR7 (p = 0.008) and vitamin D (p < 0.001) and increased total-cholesterol (p = 0.025) being found in winter/spring. Increasing vitamin D upon TNFα-blockade paralleled RA clinical improvement (r = −0.610, p = 0.027) and DHCR7 elevation (r = 0.766, p = 0.002). In conclusion, vitamin D-related polymorphisms and DHCR7 are pivotal to understand the complex, seasonal associations between vitamin D and lipid profile in RA.

## Introduction

Vitamin D is a well-known determinant of health. Apart from its classical functions in bone homeostasis, a number of physiological mechanisms have been attributed to vitamin D, including several immune-regulatory actions^1,2^. Moreover, vitamin D deficiency has been related to a wide range of disorders, from autoimmunity to cardiovascular disease (CVD)^3^.

In the field of rheumatic disorders, vitamin D deficiency is a common laboratory finding^4,5^. Based on its immune-regulatory properties, several studies have been conducted to explore the potential role of vitamin D in rheumatic conditions, such as rheumatoid arthritis (RA). Currently, its deficiency has been associated with worse disease outcomes^6–8^, however, contradictory results have been published thereafter^9^. A similar controversy exists regarding the effect of vitamin D supplementation in RA^9^. The circulating levels of vitamin D metabolites are influenced by several factors ranging from seasonality^10^ to a number of bio-activating enzymes^11^. Circulating vitamin D levels, measured by 25(OH)-vitamin D levels, are influenced by seasonality and two bioactivating cytochrome P450 isoforms, CYP27A1 and mainly CYP2R1. The next bioactivation step rendering the vitamin D hormone is catalyzed by CYP27B1, an enzyme present in numerous cell types. Moreover, the vitamin D hormone exerts its actions via Vitamin D Receptor (VDR), whose activity can be also modulated. Genetic polymorphisms have been described for all these genes, linked to altered serum levels of vitamin D metabolites (CYP2R1, CYP27B1, CYP24A1 and others) and actions (like those in VDR)^12^. Recent evidence also discloses an association between vitamin D-related polymorphisms and RA susceptibility^13^ but not severity^14^. Whether in RA these polymorphisms influence the association between vitamin D levels and clinical features and account for the heterogeneous results previously reported remains unknown.

Cohort studies confirmed a protective effect of vitamin D on CVD^15,16^, which was attributed to a favorable effect on serum lipids^17,18^. However, further studies and meta-analyses have challenged this hypothesis^19,20^. Again, additional mediators of the vitamin D or cholesterol pathways need to be considered to gain some insight into this situation. It is important to consider that both vitamin D and cholesterol synthesis share a common metabolic substrate, 7-dehydrocholesterol (7-DHC). Recently, the enzyme 7-dehydrocholesterol reductase (DHCR7), essential for the Kandutsch-Russell cholesterol synthesis, has been identified as a critical determinant of vitamin D levels in a sunlight-dependent manner^21^. In RA, where both lipid profile and vitamin D are usually altered, this enzyme could play an important role and may represent a promising therapeutic target.

We hypothesize that in RA, vitamin D-related polymorphisms and DHCR7 levels influence the relationship between vitamin D levels, lipid profile and clinical outcomes. Therefore, the main aims of the present study are (i) to evaluate the associations between vitamin D levels and clinical outcomes and lipid profile in RA patients depending on their genetic status of VDR (rs2228570), CYP27A1 (rs933994), CYP2R1 (rs10741657) and DHCR7 (rs12785878) polymorphisms, (ii) to analyze the levels of DHCR7 and its associations with the lipid profile and (iii) to prospectively evaluate the effect of RA activity on the former associations in a group of patients followed-up upon Tumor Necrosis Factor alpha (TNFα)-blockade.

## Results

### Vitamin D and RA: association with clinical features and effect of vitamin D-related polymorphisms

Serum 25(OH)-vitamin D levels were measured in 211 RA patients and 94 healthy controls (HC) (Table 1). Decreased serum levels were found in RA compared to HC [median (Q1–Q3): 23.32 (16.34–33.72) vs 29.79 (24.27–35.54) ng/ml, p < 0.001]. RA patients sampled in winter/spring exhibited decreased vitamin D levels compared to those with summer/autumn withdrawals (20.96(12.42) vs 32.42(25.25) ng/ml, p < 0.001). A similar, non-significant effect was observed in HC (29.05(12.45) vs 33.40(12.17) ng/ml). The four vitamin D-related single nucleotide polymorphisms (SNPs) showed a similar distribution between RA and HC (Supplementary Table 1). Only CYP2R1-rs10741657 and DHCR7-rs12785878 had an effect on vitamin D levels in RA patients (Supplementary Table 2).

**Table 1:** Characteristics of the study participants.

	HC (n = 94)	RA (n = 211)	p-value
***Demographical features***			
Age, years; median (range)	53.25 (27.00–83.00)	53.87 (19.00–87.81)	0.546
Gender, f/m	74/20	147/37	0.439
Sampling season, winter + spring/summer + autumn	59/35	150/61	0.148
***Blood lipids***, ***mean*** ± ***SD***			
Total-cholesterol, mg/dl	203.49 ± 33.92	207.08 ± 35.52	0.464
HDL-cholesterol, mg/dl	62.36 ± 13.42	60.33 ± 17.09	0.527
LDL-cholesterol, mg/dl	123.08 ± 30.56	122.63 ± 32.47	0.791
Total-/HDL-cholesterol ratio	3.46 ± 0.91	3.72 ± 1.36	0.310
Triglycerides, mg/dl	92.43 ± 21.23	101.35 ± 40.54	0.135
***Disease features***			
Disease duration, years		2.75 (5.34)	
Age at diagnosis, years; median (range)		49.88 (18.00–85.00)	
Disease activity (DAS28)		3.70 (2.20)	
Tender Joint Count		3.00 (7.00)	
Swollen Joint Count		1.00 (4.00)	
Patient Global Assessment (0–100)		40.00 (41.00)	
ESR, mm/h		18.00 (23.00)	
CRP, mg/dl		0.20 (0.41)	
HAQ (0–3)		0.87 (1.25)	
Pain assessment (0–10)		4.00 (4.00)	
RF (+), n(%)		118 (55.9)	
ACPA (+), n(%)		120 (56.8)	
Erosive disease (n = 129), n(%)		51 (24.1)	
***Traditional CV risk factors***, ***n***(***%***)			
Hypertension		65 (30.8)	
Dyslipidemia		52 (24.6)	
Diabetes		22 (10.4)	
Smoking		75 (35.5)	
BMI, mean ± SD		26.55 ± 4.76	
History of previous CVD		38 (18.00)	
***Treatments***, ***n***(***%***)			
None		47 (22.2)	
Glucocorticoids		104 (49.2)	
Methotrexate		141 (66.8)	
TNFα blockers		50 (23.6)	
Tocilizumab		12 (5.6)	
Vitamin D supplements		37 (17.5)	

Variables were summarized as median (IQR), mean ± SD or n(%). Statistical differences were assessed by Mann-Withney U or χ2 tests, as appropriate.

Vitamin D levels were not correlated with disease activity score (DAS28) (r = 0.047, p = 0.523), duration (r = 0.120, p = 0.090), Health Assessment Questionnaire (HAQ) (r = −0.032, 0.673), patient global assessment (r = 0.022, p = 0.774) or pain scores (r = −0.033, p = 0.659). Similarly, treatments were not related to vitamin D levels (Table 2). In the whole RA population, vitamin D deficiency (<20 ng/ml) was associated with lower HDL-cholesterol levels, winter/spring withdrawals and younger age (Table 3), but no effect was observed on disease features. Interestingly, the VDR-rs2228570 polymorphism influenced the association between vitamin D and disease outcomes. Multivariate regression analyses adjusted for age, gender, seasonality, disease activity and treatments (usage of Disease-Modifying Antirheumatic Drugs (DMARDs) and vitamin D supplements) revealed that vitamin D deficiency was an independent predictor of higher affected joint counts, disease activity and HAQ scores in patients harboring the AG/AA-genotype (Table 4A). Importantly, no difference in the prevalence of vitamin D deficiency was found between both genotypes [GG: 27/71 (38.0)% vs AG/AA: 39/123 (31.7%), p = 0.371].

**Table 2:** Effect of treatments on vitamin D levels in RA patients.

	Patients untreated	Patients treated	p-values
Glucocorticoids	22.89 (17.52)	23.73 (17.61)	0.916
Methotrexate	21.12 (15.20)	24.93 (16.18)	0.389
TNFα blockers	23.14 (17.50)	26.67 (22.48)	0.438
Tocilizumab	22.02 (10.00)	23.97 (23.52)	0.249
Vitamin D supplements	22.23 (14.55)	29.94 (39.52)	0.035

Comparison of the serum levels of 25(OH)-vitamin D (median (interquartile range)) between RA patients untreated and treated with different therapies. Differences were assessed by Mann-Withney U tests.

**Table 3:** Effect of vitamin D deficiency on demographic characteristics, lipid profile and clinical features in RA patients.

	25(OH)-vitamin D		
	<20 ng/ml (n = 69)	>20 ng/ml (n = 142)	p-value
***Demographical features***			
Age, years; median (range)	51.83 (27.00–87.81)	55.33 (19.00–87.00)	0.010
Gender, f/m	53/16	121/21	0.132
Sampling season, winter + spring/summer + autumn	57/12	93/49	0.010
***Blood lipids***, ***mean*** ± ***SD***			
Total-cholesterol, mg/dl	203.72 ± 31.69	208.63 ± 37.18	0.523
HDL-cholesterol, mg/dl	50.30 ± 17.01	62.71 ± 16.67	0.003
LDL-cholesterol, mg/dl	120.81 ± 27.28	123.45 ± 34.65	0.795
Total-/HDL-cholesterol ratio	4.03 ± 1.45	3.57 ± 1.30	0.021
Triglycerides, mg/dl	103.00 ± 56.23	99.75 ± 65.54	0.494
***Disease features***			
Disease duration, years	2.16 (4.87)	3.00 (5.31)	0.079
Age at diagnosis, years; median (range)	48.73 (18.00–81.51)	51.86 (18.00–87.81)	0.068
Disease activity (DAS28)	3.79 (2.31)	3.68 (2.13)	0.608
Tender Joint Count	3.00 (7.00)	3.00 (8.00)	0.818
Swollen Joint Count	2.00 (5.00)	1.00 (4.00)	0.962
Patient Global Assessment (0–100)	40.00 (42.00)	38.50 (41.00)	0.591
ESR, mm/h	17.00 (23.00)	18.00 (23.00)	0.924
CRP, mg/dl	0.20 (0.54)	0.20 (0.40)	0.853
HAQ (0–3)	1.00 (1.13)	0.75 (1.19)	0.235
Pain assessment (0–10)	4.30 (4.00)	4.00 (4.00)	0.352
RF (+), n(%)	41 (59.4)	77 (54.2)	0.367
ACPA (+), n(%)	40 (57.9)	40 (56.3)	0.745
Erosive disease (n = 129), n(%)	20 (28.9)	30 (36.5)	0.282
***Traditional CV risk factors***, ***n***(***%***)			
Hypertension	19 (27.5)	46 (32.3)	0.535
Dyslipidemia	20 (28.9)	32 (22.5)	0.396
Diabetes	9 (13.0)	13 (9.1)	0.376
Smoking	30 (43.4)	45 (31.6)	0.315
BMI, mean ± SD	28.30 ± 5.17	26.53 ± 4.40	0.089
History of previous CVD	17 (24.6)	21 (14.7)	0.080
***Treatments***, ***n***(***%***)			
None	20 (28.9)	27 (19.0)	0.101
Glucocorticoids	33 (47.8)	71 (50.0)	0.767
Methotrexate	43 (62.3)	98 (69.0)	0.365
TNFα blockers	14 (20.2)	36 (25.3)	0.402
Tocilizumab	5 (7.2)	7 (4.9)	0.751
Vitamin D supplements	9 (13.0)	(19.7)	0.232

Comparison of demographic characteristics, lipid profiles and clinical features in RA patients according to vitamin D deficiency status (defined as 25(OH)-vitamin D < 20 ng/ml). Variables were summarized as median (IQR), mean ± SD or n(%). Statistical differences were assessed by Mann-Withney U or χ2 tests, as appropriate.

**Table 4:** Effect of vitamin D deficiency on clinical features and lipid profile depending on VDR rs2228570 status.

	GG (n = 71)		AG/AA (n = 123)	
	Mean difference (95% CI)	p-value	Mean difference (95% CI)	p-value
***A) Clinical features***				
Disease activity (DAS28)	−0.291 (−1.075, 0.492)	0.466	0.704 (0.126, 1.281)	0.018
Tender Joint Count	−0.243 (−5.420, 0.570)	0.112	2.92 (0.890, 4.930)	0.005
Swollen Joint Count	−1.320 (−3.250, 0.620)	0.182	1.960 (0.73, 3.190)	0.002
Patient Global Assessment	−2.490 (−15.820, 10.820)	0.714	6.970 (−3.210, 17.150)	0.179
ESR	2.702 (−7.325, 12.736)	0.597	1.716 (−7.868, 11.302)	0.726
CRP	0.780 (−0.382, 0.538)	0.740	−0.016 (−0.322, 0.354)	0.925
HAQ	0.012 (−0.363, 0.387)	0.950	0.306 (0.041, 0.572)	0.024
Pain assessment	−1.260 (−3.350, 0.840)	0.240	0.890 (−0.140, 1.930)	0.092
***B) Lipid profile***				
Total-cholesterol	−11.491 (−29.913, 6.930)	0.221	3.538 (−9.727, 16.803)	0.601
HDL-cholesterol	−5.045 (−13.923, 3.832)	0.265	−7.363 (−13.936, −0.790)	0.028
LDL-cholesterol	−7.36 (−24.324, 9.592)	0.395	0.429 (−12.72, 13.568)	0.949
Total-/HDL-cholesterol ratio	0.283 (−0.330, 0.890)	0.365	0.575 (0.098, 1.052)	0.018
Triglycerides	−28.75 (−82.328, 24.827)	0.293	68.732 (0.566, 136.897)	0.048

The effect of vitamin D deficiency was analyzed by multiple linear regression analyses adjusted by age, gender, seasonality, vitamin D supplementation and DMARD usage (glucocorticoids, methotrexate, TNFα-blockers and tocilizumab). Mean differences and 95% CI were computed for each parameter. Mean differences were referred to the comparison between vitamin D deficiency (<20 ng/ml) vs. no deficiency (>20 ng/ml).

### Vitamin D and lipid profiles in RA

Next, the associations between vitamin D and lipid profiles were analyzed. Vitamin D levels were correlated to those of HDL-cholesterol (r = 0.217, p < 0.001) in RA patients. Equivalent results were obtained when the total-/HDL-cholesterol ratio^22^ was analyzed (r = −0.227, p = 0.004).

**Figure 1:**
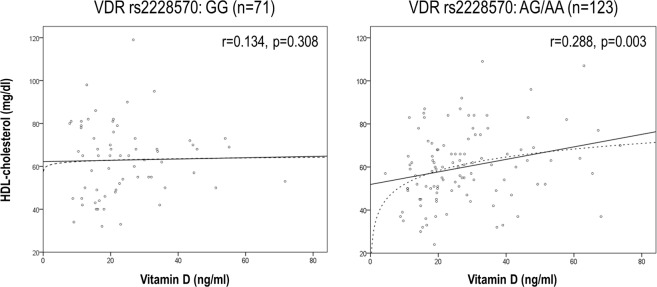
Association between HDL-cholesterol and vitamin D serum levels in RA patients. RA patients were stratified depending on their VDR rs2228570 and the correlation between HDL-cholesterol and vitamin D serum levels was assessed by Spearman rank’s test and linear regression analyses (solid lines). In patients with the AG/AA-status, a logarithmic model (dashed line) showed a good fitting.

Importantly, the VDR-rs2228570 polymorphism influenced the association between vitamin D and HDL-cholesterol levels: whereas a positive correlation was observed for patients exhibiting the AG or AA genotype, it was absent in their GG-counterparts (Fig. 1). Multivariate regression analyses adjusted for age, gender, seasonality, disease activity and treatments (usage of DMARDs and vitamin D supplements) confirmed that vitamin D was an independent predictor of HDL levels (B[95% CI], p: 0.246[0.036, 0.455], p = 0.022) in patients harboring the AG/AA genotype, hence ruling out a potential contribution of disease activity and thus, a reverse causality phenomenon. Importantly, this association remained after adjusting for body mass index (BMI) (0.145[0.044, 1.861], p = 0.043), hence ruling out a major effect of obesity in the association between vitamin D and lipid profile. Again, equivalent results were obtained with the total-/HDL-cholesterol ratio. Exclusion of patients taking vitamin D supplements from the analysis did not change these associations. Moreover, vitamin D deficiency showed a different clinical outcome depending on the VDR-rs2227850 status, being an independent predictor of a detrimental lipid profile in AG/AA-patients (Table 4B), but not in their GG-counterparts. Again, adjusting for disease activity did not change the association between vitamin D deficiency and lipid profile. The rest of the polymorphisms studied did not exhibit such an effect.

Taken together, our results suggest that vitamin D levels showed different associations with the lipid profile and clinical features depending on the VDR-rs2227850 status.

### DHCR7 in the interplay among seasonality, vitamin D and lipid profile in RA

Next, the potential role for DHCR7, highly relevant in the connection between cholesterol and vitamin D synthesis, was analyzed.

DHCR7 serum levels were decreased in RA compared to HC in individuals sampled in winter/spring (519.95(581.35) vs 718.86(642.46) pg/ml, p = 0.012) but not in summer/autumn (856.29(1126.34) vs 783.54(512.23) pg/ml, p = 0.354). Although a clear effect of the seasonality on DHCR7 levels was observed in RA (winter/spring: 519.95(581.35) vs summer/autumn: 856.29(1126.34) pg/ml, p < 0.001), the same could not be applied to the control group (winter/spring: 698.86(642.46) vs summer/autumn: 738.54(512.53) pg/ml, p = 0.719). Moreover, DHCR7-rs1287858 variants were associated with DHCR7 serum levels (Supplementary Fig. 1). Interestingly, RA patients with a history of previous CVD exhibited lower DHCR7 serum levels than their CVD-free counterparts (411.35(382.03) vs 621.68(713.33), p = 0.024). DHCR7 levels were not associated with BMI (r = 0.117, p = 0.240).

The associations among DHCR7, vitamin D and HDL-cholesterol levels followed a seasonal pattern. Vitamin D was strongly correlated with HDL-cholesterol levels (r = 0.354, p = 0.009) and total-/HDL-cholesterol ratio (r = −0.393, p = 0.003) in summer/autumn; whereas only a weak correlation with HDL-cholesterol (r = 0.215, p = 0.014) was found in winter/spring. Stratification of patients by their DCHR7-rs12785878 status revealed that this correlation remained in individuals with the TT/TG genotype (r = 0.205, p = 0.017) during this season, but were absent in their GG-counterparts (r = 0.252, p = 0.346). On the other hand, vitamin D levels paralleled those of DHCR7 in summer/autumn (r = 0.441, p = 0.004), suggesting a balance that is lost in winter/spring samples (r = 0.028, p = 0.775). Remarkably, decreased DHCR7 was associated with age at onset (r = −0.301, p = 0.009) and Rheumatoid Factor (RF) titre (r = −0.326, p = 0.014) in winter/spring. It is interesting to note that total-cholesterol levels were higher in winter/spring samples than in those obtained in summer/autumn (214.74 ± 37.52 vs 188.36 ± 40.00 mg/dl, p = 0.025), thus confirming seasonal variations like those observed for vitamin D and DHCR7.

Next, the influence of RA disease activity on these associations was studied. Although DHCR7 was positively correlated (moderate effect) to vitamin D levels in the whole RA group (r = 0.209, p = 0.011), when patients were stratified according to disease activity, this association only remained in the low disease activity group (strong effect) (DAS28 ≤ 2.6, n = 35: r = 0.407, p = 0.015), whereas no association was observed in patients with high disease activity. No differences in the frequency of vitamin D deficiency was noted between groups (p = 0.738).

Overall, these results support that altered DHCR7 levels are pivotal to understand the imbalance between vitamin D and lipid profile in RA. Moreover, seasonal variations and clinical features are key to explain the altered DHCR7 levels.

### Vitamin D, DHCR7 and lipid profiles in RA: a prospective study

Since our previous findings point to an effect of disease activity on the association between vitamin D and the lipid profile in RA, we conducted a prospective study to shed new light on the associations among these mediators upon anti-TNFα-mediated disease activity control. To this end, a subgroup of 13 biological-naïve RA patients was followed-up for 3 months and serum samples were obtained at baseline (BL) and after treatment (post-treatment, PT).

**Figure 2:**
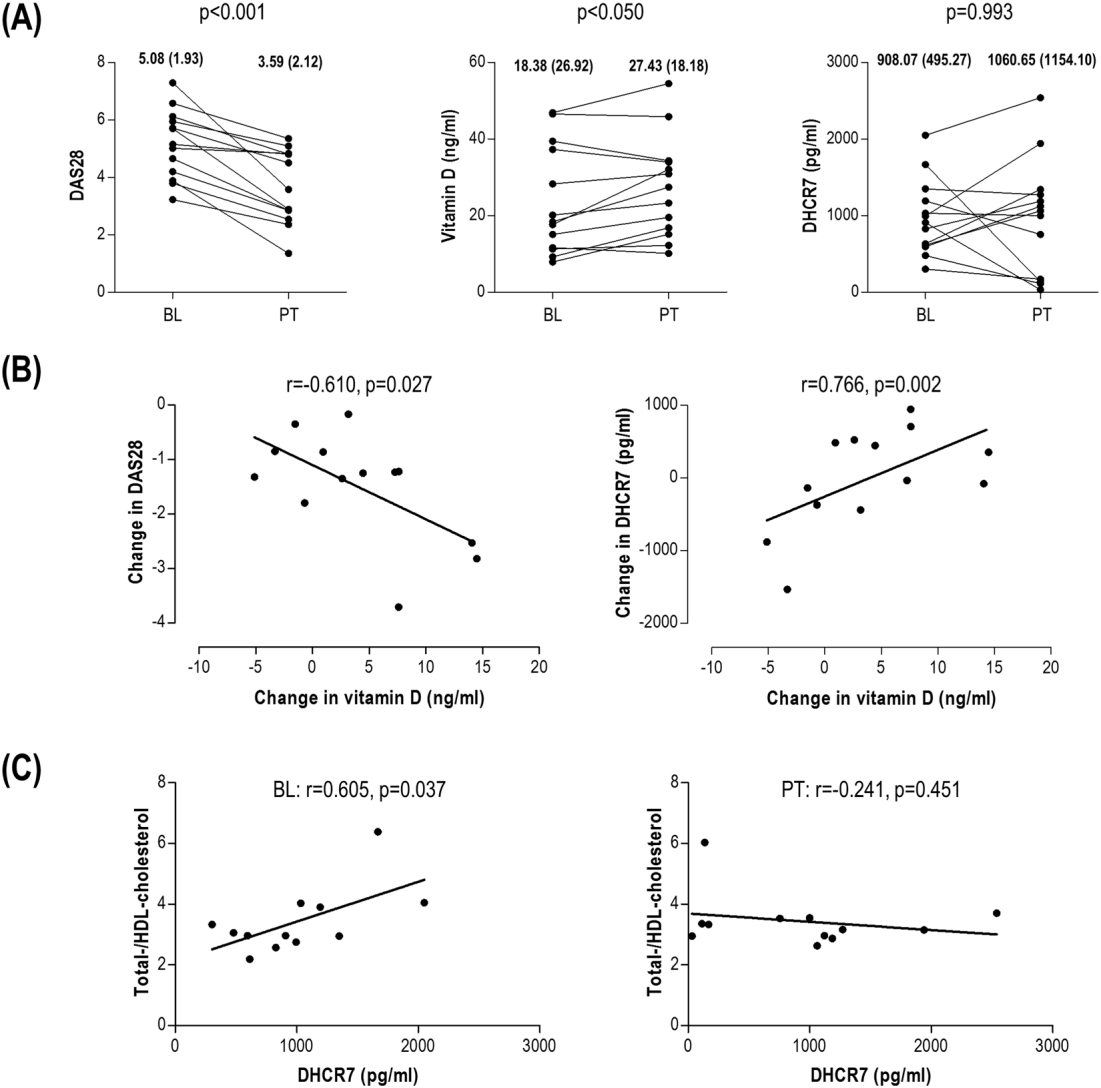
Vitamin D, DHCR7 and lipid profiles upon TNFa-blockade in RA. (**A**) Changes in DAS28, vitamin D and DHCR7 in patients following TNFa-blockade therapy for 3 months. Values before the initiation of the treatment (baseline, BL) and after treatment (post-treatment, PT) were evaluated by Wilcoxon paired tests. BL and PT medians (interquartile range) for each of the variables analyzed was included at the top of each graph. (**B**) Associations between the change (PT minus BL) in serum vitamin D levels and DAS28 as well as in DHCR7 serum levels. (**C**) Correlation between the total-/HDL-cholesterol ratio and DHCR7 serum levels before (BL) and after (PT) treatment. Correlations were evaluated by Spearman rank’s tests.

Expectedly, DAS28 dropped after treatment (Fig. 2A). TNFα-blockade resulted in an increase in vitamin D levels, but no effect was observed on DHCR7 (Fig. 2A). A positive strong association between the change in DAS28 and that of vitamin D was noted, hence indicating that the higher the reduction in DAS28, the greater the increase in vitamin D (Fig. 2B). Moreover, the change in vitamin D levels strongly paralleled that of DHCR7 (Fig. 2B), although no absolute changes were observed in the latter. DHCR7 exhibited a strong positive correlation with total-/HDL-cholesterol ratio at baseline, whereas this correlation was not observed after treatment (Fig. 2C). All these changes were observed even in the short-term and with no changes in BMI, hence reinforcing the association between vitamin D, lipid profile and DHCR7 independently of BMI.

All these results suggest that increasing vitamin D levels can be observed upon TNFα-blockade, in association with the clinical response. Additionally, whereas DHCR7 may exhibit an association with a detrimental lipid profile under high disease activity states, this situation can be counteracted by an adequate disease activity control. Indeed, a parallel increase in DHCR7 serum levels and vitamin D was observed upon treatment.

## Discussion

Although the role of vitamin D in RA has been the object of profound research during last decades, a number of unresolved questions remains. Most of the studies have focused on the levels of vitamin D alone, which only provides a limited perspective of the whole picture of the vitamin D relevance. Moreover, little attention has been paid to the potential association between vitamin D and lipid profiles as well as comorbidities in RA. In the present manuscript, we demonstrate for the first time that the effect of vitamin D levels on the lipid profile and clinical features in RA patients strongly depends not only on VDR genetic polymorphisms but also on DHCR7 regulation of vitamin D and cholesterol synthesis, hence pointing to the involvement of additional mediators to account for the effects of vitamin D in RA.

Our findings revealed that vitamin D-related polymorphisms have an important impact in the association between vitamin D levels and lipid profile and clinical features in RA. An enormous controversy exists regarding the association of vitamin D with clinical outcomes in RA (reviewed in)^9^. Genetic polymorphisms may be key to understand the heterogeneity observed on vitamin D levels and actions regarding clinical features. Indeed, since a differential distribution of vitamin D-related polymorphisms was observed among different ethnic populations and latitudes, they may be conceived as a source of heterogeneity among studies. Among all the polymorphisms herein analyzed, VDR-rs2227850 exhibited the strongest effect. This is in line with the central role of VDR for vitamin D actions, clearly illustrated by studies with VDR knock-out models^19^. In our study, individuals with the AG/AA status showed an impaired lipid profile and aggravated clinical features upon vitamin D deficiency, whereas no effect was observed in GG-carriers. Early studies demonstrated a functional impairment of the VDR molecule originated from the A allele^23,24^, linked to a lower intracellular signaling^25^. Recently, a reduced VDR activity has been associated with an increased cholesterol biosynthesis *in vitro*, due to a lower inhibition of the HMG-CoA reductase^26^. Moreover, VDR knock-out mice exhibit higher cholesterol levels compared to their wild-type littermates^27^. These pieces of evidence may account for our findings on the effect of VDR-rs2227850 status on RA patients.

The magnitude of VDR signals depends upon the amount of circulating vitamin D, since the binding of active vitamin D metabolites to the VDR favors its stabilization, whereas VDR degradation is increased at low vitamin D levels^28–30^. Therefore, the increased VDR turnover induced by low vitamin D levels may aggravate the adverse cellular effects of vitamin D deficiency. Additionally, 25(OH)-vitamin D synergizes with vitamin D hormone for signals downstream VDR activation^31^. This may explain the non-linear association observed in carriers of the A allele between vitamin D and HDL at low vitamin D levels. Importantly, no differences were observed in the polymorphisms of the activating enzymes of vitamin D (CYP2R1 and CYP27A1). Then, a differential sensitivity to vitamin D depending on genetic variants of both VDR and vitamin D catabolic machinery may underlie this effect. Interestingly, rheumatic conditions have been reported to be more refractory to vitamin D actions than the responses to vitamin D in non-rheumatic individuals^32^, and attributed to an impaired vitamin D metabolism. These notions may have important implications for vitamin D supplementation.

A number of clinical studies have failed to demonstrate and adequate clinical effect of vitamin D supplementation in RA. However, in these trials, only one out of three patients reached adequate levels (reviewed in)^9,33^. Regardless of the contribution of vitamin D refractoriness or other clinical reasons to the reported findings, a better stratification of RA patients undergoing vitamin D supplementation may improve these outcomes. It has been proposed that novel biomarkers are needed to guide vitamin D supplementation^32^. Based on our findings, VDR status or HDL levels may be useful in this scenario.

**Figure 3:**
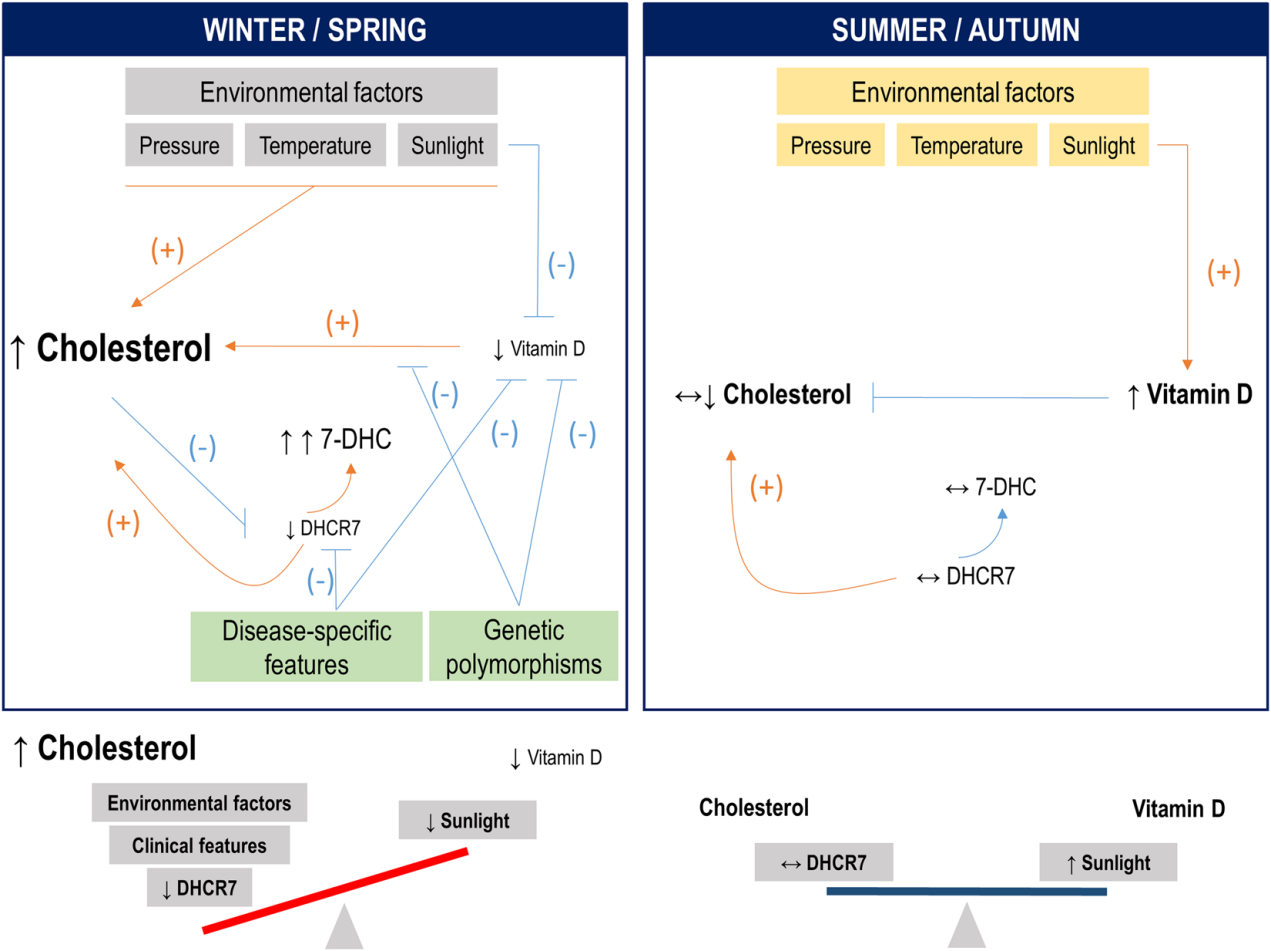
Overview model of the interplay of vitamin D and lipid profiles in RA. Our results support a role for DHCR7 and genetic polymorphisms to account for the associations between vitamin D and lipid profiles in RA, in a seasonal-dependent manner. In winter/spring, environmental factors can trigger an imbalanced vitamin D/lipid profile, which can be in turn exacerbated by their mutual, complex regulation mechanisms, decreasing DHCR7 levels playing a crucial role. Due to the decreased DHCR7 levels and low sunlight exposure, the common substrate 7-DHC may be mainly shunted towards cholesterol rather than vitamin D production, hence aggravating the imbalance. Disease-specific features and genetic polymorphisms can reinforce these pathogenic loops. In summer/autumn, a more favorable effect on vitamin D by environmental factors may lead to a tighter balance among vitamin D, lipid profile and DHCR7 levels, as demonstrated by the positive associations observed in our study. (−) denotes a negative/inhibition effect, whereas (+) denotes a promoting effect. ↑: increased; ↔ : no change, normal levels; ↓: decreased.

A remarkable finding from our study was the role of DHCR7 levels in RA and its association with the lipid profile, vitamin D and CVD disease. Despite exhibiting a central role to control circulating vitamin D levels, little attention has been paid to DHCR7, and its role in RA and rheumatic diseases in relation to lipid profile and vitamin D had not been previously explored. Varying levels of DHCR7 and sunlight exposure ultimately control both vitamin D and cholesterol synthesis^21^. Our findings suggest a role of DHCR7 to understand the connections between vitamin D and lipid profile in a seasonal-dependent manner, also with the involvement of clinical features. Environmental factors in winter/spring are known to produce seasonal increases in cholesterol levels^34–36^. High cholesterol levels are known to promote DHCR7 proteasomal degradation and suppress DHCR7 expression via SREBP-2^37^, hence leading to a 7-DHC accumulation. However, low sunlight impedes the conversion of the under-utilized 7-DHC to vitamin D, hence causing decreased vitamin D levels, which can in turn result in increased cholesterol levels by promoting a less efficient negative control of cholesterol synthesis via HMGCoA reductase^38^, thus aggravating the cholesterol/vitamin D imbalance (Fig. 3) and explaining the seasonal decrease of DHCR7 in our study in winter/spring sample. Moreover, disease-related features were also negatively associated with DHCR7 levels, hence strengthening this pathogenic loop. The accumulation of 7-DHC can also lead to the production of harmful metabolites^39^. On the contrary, lower cholesterol levels are found during summer/autumn, in addition to higher vitamin D levels due to the increased sunlight. Then, vitamin D can negatively regulate cholesterol levels via HMGCoA reductase inhibition and also by suppressing DHCR7 activity (but not levels)^40^. Under these circumstances, proteasomal-mediated DHCR7 degradation is not enhanced and DHCR7 does not represent a limiting factor. In this setting, it is tempting to speculate that a balanced cholesterol/vitamin D production is achieved (Fig. 3), in line with the positive correlations among these mediators observed in our study, which were absent in the low-sunlight period. Moreover, these associations showed a rs12785878-driven effect, since patients with genetically-determined higher DHCR7 serum levels seem to counteract/be protected against the seasonal-associated DHCR7 decrease. Taken together, these observations strongly confirm a role for genetic determinants in the association between vitamin D and lipid profile.

It is interesting to consider that the picture observed in winter/spring (impaired lipid profile, low vitamin D levels, low DHCR7, increased 7-DHC and lack of association DHCR7/vitamin D), together with the seasonal aggravation of the disease^41^ may render these individuals more likely to develop CVD. Indeed, a seasonal pattern for CVD occurrence is well documented^42,43^. The low levels of DHCR7 in patients with a history of previous CVD is in line with these facts. Overall, these lines of evidence raise the question of whether seasonal variations need to be considered for decision making in the clinical setting. Although a growing number of biomarkers are being reported to guide the clinician in the era of personalized medicine, environmental factors may indeed represent additional, promising candidates to be considered in this framework.

Finally, disease burden may be an additional factor that account for some controversy observed in previous studies. Our prospective analyses revealed different associations among vitamin D, lipid profile and DHCR7 before and after disease activity control by TNFα-blockade. Decreasing TNFα serum levels can lead to a reduced expression of vitamin D-catabolic enzyme (CYP24A1)^44,45^, which can explain the increasing 25(OH)-vitamin D levels found after anti-TNFα treatment. Moreover, abrogation of inflammation may reduce vitamin D consumption by immune cells, also leading to higher circulating levels. Either the underlying mechanism, the change in vitamin D upon treatment paralleled that of DHCR7. This finding aligns with the positive association between both mediators in patients under remission states, but not in those with high disease activity observed in the cross-sectional study. Moreover, the detrimental association between DHCR7 and lipid profile is abolished after therapy. Therefore, DHCR7 may play a key role in the beneficial effects of TNFα-blockade on lipid profiles in RA^46,47^. Larger and long-term prospective studies are warranted to evaluate the effect on vitamin D fluctuations on DHCR7 and lipid profiles.

In conclusion, our results revealed complex associations between vitamin D and the lipid profile as well as clinical features in RA, VDR polymorphism and DHCR7 playing a pivotal role. These findings shed new light into the potential effects of vitamin D beyond bone metabolism in RA, including lipid profile homeostasis and CVD, independently of BMI. On the other hand, our conclusions reinforce the idea that vitamin D levels alone may provide a limited clinically relevant information, hence emphasizing the need of considering additional biomarkers in both the clinical setting and research studies. However, the present study has some limitations that must be remarked, such as the cross-sectional design, which did not allow to establish causality among the conclusions achieved, and the lack of information about dietary determinants of vitamin D levels. Furthermore, the genetic associations and the results observed in the prospective study need replication in larger cohorts. Moreover, despite DHCR7 serum levels were observed to be altered in RA patients, whether the same could be applied to DHCR7 enzymatic activity remains unknown and it proves this field worthy of further research.

## Material and Methods

### Ethics statement

Approval for the study was obtained from the Institutional Review Board (Comité de Ética Regional de Investigación Clínica, reference PI16/00113), in compliance with the Declaration of Helsinki. All participants gave a written informed consent prior to their inclusion in the study.

### Patients

Our study involved three populations: (1) a group of 211 RA patients (2010 ACR/EULAR classification criteria) enrolled from the Department of Rheumatology at Hospital Universitario Central de Asturias, (2) a group of 13 biological-naïve RA patients (12 women, median age 43 (range: 30–65), DAS28 5.08(1.93), 38.5% RF+, 46.1% ACPA+), candidates for TNFα-blockers prospectively followed-up for 3 months and (3) a group of 94 age- and gender-matched healthy volunteers (healthy controls, HC) recruited from the same population. Inclusion criteria were (i) fulfill the 2010 EULAR/ACR classification criteria, (ii) not been diagnosed with another immune-mediated systemic rheumatic disease at the time of recruitment, (iii) been clinically managed and treated according to EULAR guidelines. Exclusion criteria were (i) recent (<3 months) infections or surgeries, (ii) cancer diagnosis or (iii) pregnancy. All subjects were recruited from the region Asturias (north Spain, main city: Oviedo (latitude 43°N)) and were of Caucasian origin. A complete clinical examination, including Disease Activity Score 28-joints (DAS28) calculation, was performed on all patients. Clinical records were examined in order to register traditional CV risk factors and history of CVD. In the prospective analysis, a blood sample was obtained immediately before (baseline, BL) as well as 3-months after initiation of TNFα-blockade therapy (post-treatment, PT). Based on a priori calculations, the sample size in this prospective branch (with a paired design) allowed the identification of a difference of 1.2 points in the DAS28 score, with a power of >0.80 (at an alpha = 0.050).

### Biochemical analyses

Automated serum lipids analysis was performed on all participants in fresh blood samples (overnight fasting) and serum samples were stored at −80 °C until other laboratory measurements were carried out. In compliance with EULAR recommendations, the total-/HDL-cholesterol ratio^22^ was calculated and analyzed as a better estimation of the lipid profile.

Serum levels of 25(OH)-vitamin D, the metabolite currently used to estimate vitamin D status, were measured using an electro-chemiluminescence binding assay (Elecsys assay, Roche), according to the manufacturer instructions (detection limit: 4.01 ng/ml). Intra- and inter-assays reproducibility yielded values of 5.5% and 10.9%, respectively. Vitamin D deficiency was defined as <20 ng/ml 25(OH)-vitamin D serum levels^6,48^.

### DHCR7 serum levels

Serum DHCR7 levels were quantified by an immunoassay (Cusabio Biotech), following the manufacturer´s protocol (detection limit: 31.25 pg/ml).

### Single nucleotide polymorphisms genotyping

SNP genotyping was performed in study subjects with DNA samples available [194(91.9%) RA patients and 88(93.6%) HC]. The genotyped population was representative from that of the whole study population (Supplementary Table 3). DNA was isolated from peripheral blood using conventional methods (hypotonic lysis). The VDR (rs2228570), CYP27A1 (rs933994), CYP2R1 (rs10741657) and DHCR7 (rs12785878) polymorphisms were genotyped with TaqMan predesigned single-nucleotide polymorphism (SNP) genotyping assays (C__12060045_20, C__2070283_20, C__2958430_10 and C__32063037_10, respectively) in a 7900 HT Real-Time polymerase chain reaction (PCR) system, as previously described^49^.

### Statistical analyses

Continuous variables were summarized as median (interquartile range) or mean ± standard deviation. Categorical variables were described as n(%). Differences between groups were analyzed by Mann Withney U or chi-squared tests. Wilcoxon test was used for paired samples. Correlations were assessed by Spearman ranks’ test. Multiple linear regression analyses were performed to evaluate the association between continuous variables adjusted for confounders. Beta (B) and 95% confidence intervals (CI) were computed. Before entering in multiple regression analyses, variables were log-transformed to achieve a normal distribution. The effect of vitamin D deficiency (<20 ng/ml) on clinical features and lipid profile was analyzed by generalized linear models adjusted for confounders (age, gender, seasonality, disease activity, DMARD usage and vitamin D supplements), and adjusted mean difference (deficiency vs. no deficiency) and 95% CI were calculated. A p-value < 0.050 was considered statistically significant. Statistical analyses were performed in SPSS 24.0 and GraphPad Prism 5.0 for Windows.

## Supplementary information

Supplementary Materials

## References

[1] Verstuyf A, Carmeliet G, Bouillon R, Mathieu C (2010). Vitamin D: a pleiotropic hormone. Kidney Int..

[2] Hewison, M. Vitamin D and the immune system: new perspectives on an old theme. *Endocrinol*. *Metab*. *Clin*. *North Am*. **39**, 365–79, table of contents (2010). 10.1016/j.ecl.2010.02.010 PMC2879394 20511058

[3] Holick MF (2012). Evidence-based D-bate on health benefits of vitamin D revisited. Dermatoendocrinol..

[4] Nikiphorou E, Uksila J, Sokka T (2018). A cross-sectional study of vitamin D levels in a large cohort of patients with rheumatic diseases. Clin. Rheumatol..

[5] Cutolo M (2008). Vitamin D or hormone D deficiency in autoimmune rheumatic diseases, including undifferentiated connective tissue disease. Arthritis Res. Ther..

[6] Kerr GS (2011). Prevalence of vitamin D insufficiency/deficiency in rheumatoid arthritis and associations with disease severity and activity. J. Rheumatol..

[7] Turhanoğlu AD (2011). The relationship between vitamin D and disease activity and functional health status in rheumatoid arthritis. Rheumatol. Int..

[8] Haque UJ, Bartlett SJ (2010). Relationships among vitamin D, disease activity, pain and disability in rheumatoid arthritis. Clin. Exp. Rheumatol..

[9] Bragazzi NL (2017). Vitamin D and rheumatoid arthritis: an ongoing mystery. Curr. Opin. Rheumatol..

[10] Cutolo M (2006). Circannual vitamin d serum levels and disease activity in rheumatoid arthritis: Northern versus Southern Europe. Clin. Exp. Rheumatol..

[11] Dusso AS, Brown AJ, Slatopolsky E (2005). Vitamin D. Am. J. Physiol. Renal Physiol..

[12] Wang TJ (2010). Common genetic determinants of vitamin D insufficiency: a genome-wide association study. Lancet (London, England).

[13] Yarwood A (2013). Enrichment of vitamin D response elements in RA-associated loci supports a role for vitamin D in the pathogenesis of RA. Genes Immun..

[14] Viatte S (2014). The role of genetic polymorphisms regulating vitamin D levels in rheumatoid arthritis outcome: a Mendelian randomisation approach. Ann. Rheum. Dis..

[15] Higgins MJ (2013). The effect of vitamin D levels on the assessment of disease activity in rheumatoid arthritis. Clin. Rheumatol..

[16] Hutchinson MS, Grimnes G, Joakimsen RM, Figenschau Y, Jorde R (2010). Low serum 25-hydroxyvitamin D levels are associated with increased all-cause mortality risk in a general population: the Tromsø study. Eur. J. Endocrinol..

[17] Jorde R, Figenschau Y, Hutchinson M, Emaus N, Grimnes G (2010). High serum 25-hydroxyvitamin D concentrations are associated with a favorable serum lipid profile. Eur. J. Clin. Nutr..

[18] Jorde R, Grimnes G (2011). Vitamin D and metabolic health with special reference to the effect of vitamin D on serum lipids. Prog. Lipid Res..

[19] Wang H, Xia N, Yang Y, Peng D-Q (2012). Influence of vitamin D supplementation on plasma lipid profiles: a meta-analysis of randomized controlled trials. Lipids Health Dis..

[20] Elamin MB (2011). Vitamin D and cardiovascular outcomes: a systematic review and meta-analysis. J. Clin. Endocrinol. Metab..

[21] Patwardhan VG, Khadilkar AV, Chiplonkar SA, Mughal ZM, Khadilkar VV (2015). Varying relationship between 25-hydroxy-vitamin D, high density lipoprotein cholesterol, and serum 7-dehydrocholesterol reductase with sunlight exposure. J. Clin. Lipidol..

[22] Agca R (2017). EULAR recommendations for cardiovascular disease risk management in patients with rheumatoid arthritis and other forms of inflammatory joint disorders: 2015/2016 update. Ann. Rheum. Dis..

[23] Arai H (1997). A vitamin D receptor gene polymorphism in the translation initiation codon: effect on protein activity and relation to bone mineral density in Japanese women. J. Bone Miner. Res..

[24] Uitterlinden AG, Fang Y, Van Meurs JBJ, Pols HAP, Van Leeuwen JPTM (2004). Genetics and biology of vitamin D receptor polymorphisms. Gene.

[25] Jurutka PW (2000). The polymorphic N terminus in human vitamin D receptor isoforms influences transcriptional activity by modulating interaction with transcription factor IIB. Mol. Endocrinol..

[26] Li S (2016). Increase of circulating cholesterol in vitamin D deficiency is linked to reduced vitamin D receptor activity via the Insig-2/SREBP-2 pathway. Mol. Nutr. Food Res..

[27] Wang J-H (2009). Serum cholesterol and expression of ApoAI, LXRbeta and SREBP2 in vitamin D receptor knock-out mice. J. Steroid Biochem. Mol. Biol..

[28] Wiese RJ, Uhland-Smith A, Ross TK, Prahl JM, DeLuca HF (1992). Up-regulation of the vitamin D receptor in response to 1,25-dihydroxyvitamin D3 results from ligand-induced stabilization. J. Biol. Chem..

[29] van den Bemd GC, Pols HA, Birkenhäger JC, van Leeuwen JP (1996). Conformational change and enhanced stabilization of the vitamin D receptor by the 1,25-dihydroxyvitamin D3 analog KH1060. Proc. Natl. Acad. Sci. USA.

[30] Arbour NC, Prahl JM, DeLuca HF (1993). Stabilization of the vitamin D receptor in rat osteosarcoma cells through the action of 1,25-dihydroxyvitamin D3. Mol. Endocrinol..

[31] Lou Y-R (2010). 25-Hydroxyvitamin D(3) is an agonistic vitamin D receptor ligand. J. Steroid Biochem. Mol. Biol..

[32] Sainaghi PP, Bellan M, Antonini G, Bellomo G, Pirisi M (2011). Unsuppressed parathyroid hormone in patients with autoimmune/inflammatory rheumatic diseases: implications for vitamin D supplementation. Rheumatology (Oxford)..

[33] Gatenby P, Lucas R, Swaminathan A (2013). Vitamin D deficiency and risk for rheumatic diseases: an update. Curr. Opin. Rheumatol..

[34] Gordon DJ (1988). Cyclic seasonal variation in plasma lipid and lipoprotein levels: the Lipid Research Clinics Coronary Primary Prevention Trial Placebo Group. J. Clin. Epidemiol..

[35] Ockene IS (2004). Seasonal variation in serum cholesterol levels: treatment implications and possible mechanisms. Arch. Intern. Med..

[36] Janecki JM (2013). Cholesterol level in human serum: seasonal variations and differences in 14 distant regions. Ann. Clin. Lab. Sci..

[37] Prabhu AV, Luu W, Sharpe LJ, Brown A (2016). J. Cholesterol-mediated Degradation of 7-Dehydrocholesterol Reductase Switches the Balance from Cholesterol to Vitamin D Synthesis. J. Biol. Chem..

[38] Gupta AK, Sexton RC, Rudney H (1989). Effect of vitamin D3 derivatives on cholesterol synthesis and HMG-CoA reductase activity in cultured cells. J. Lipid Res..

[39] Prabhu AV, Luu W, Li D, Sharpe LJ, Brown A (2016). J. DHCR7: A vital enzyme switch between cholesterol and vitamin D production. Prog. Lipid Res..

[40] Zou L, Porter TD (2015). Rapid suppression of 7-dehydrocholesterol reductase activity in keratinocytes by vitamin D. J. Steroid Biochem. Mol. Biol..

[41] Watad A (2017). Seasonality and autoimmune diseases: The contribution of the four seasons to the mosaic of autoimmunity. J. Autoimmun..

[42] Cheng TO (2005). Seasonal variation in serum cholesterol levels may be another explanation for seasonal variation in acute myocardial infarction. Int. J. Cardiol..

[43] Manfredini, R. & Boari, B. Seasonal variation in serum cholesterol levels: another piece in the puzzle of the winter excess of cardiovascular deaths? *Arch*. *Intern*. *Med*. **164**, 2505–6; author reply 2506–7 (2004). 10.1001/archinte.164.22.2505-b 15596653

[44] Hummel DM, Fetahu IS, Gröschel C, Manhardt T, Kállay E (2014). Role of proinflammatory cytokines on expression of vitamin D metabolism and target genes in colon cancer cells. J. Steroid Biochem. Mol. Biol..

[45] Ziv E, Koren R, Zahalka MA, Ravid A (2016). TNF-α increases the expression and activity of vitamin D receptor in keratinocytes: role of c-Jun N-terminal kinase. Dermatoendocrinol..

[46] Choy E, Sattar N (2009). Interpreting lipid levels in the context of high-grade inflammatory states with a focus on rheumatoid arthritis: a challenge to conventional cardiovascular risk actions. Ann. Rheum. Dis..

[47] Robertson J, Peters MJ, McInnes IB, Sattar N (2013). Changes in lipid levels with inflammation and therapy in RA: a maturing paradigm. Nat. Rev. Rheumatol..

[48] Urruticoechea-Arana A (2015). Vitamin D deficiency in chronic inflammatory rheumatic diseases: results of the cardiovascular in rheumatology [CARMA] study. Arthritis Res. Ther..

[49] Rodríguez-Carrio J (2017). High triglycerides and low high-density lipoprotein cholesterol lipid profile in rheumatoid arthritis: A potential link among inflammation, oxidative status, and dysfunctional high-density lipoprotein. J. Clin. Lipidol..

