# Supplementary material for: Vitamin D Receptor Polymorphism and DHCR7 Contribute to the Abnormal Interplay Between Vitamin D and Lipid Profile in Rheumatoid Arthritis
Source: Sci Rep. 2019 Feb 22;9:2546. doi: 10.1038/s41598-019-38756-8 (PMC6385268; doi:10.1038/s41598-019-38756-8)

**SUPPLEMENTARY MATERIAL**

**VITAMIN D RECEPTOR POLYMORPHISM AND DHCR7 CONTRIBUTE TO THE ABNORMAL INTERPLAY BETWEEN VITAMIN D AND LIPID PROFILE IN RHEUMATOID ARTHRITIS**

Javier Rodríguez-Carrio^1,2,3^, Mercedes Alperi-López^3,4^, Manuel Naves-Díaz^2,3^, Adriana Dusso^2,3^, Patricia López^1,3^, Francisco Javier Ballina-García^3,4^, Jorge B. Cannata-Andía^2,3#^, Ana Suárez^1,3^

^1^Area of Immunology, Department of Functional Biology, University of Oviedo, Oviedo, Spain

^2^ Bone and Mineral Research Unit, REDinREN del ISCIII, Hospital Universitario Central de Asturias, Oviedo, Spain

^3^ Instituto de Investigación Sanitaria del Principado de Asturias (ISPA), Oviedo, Spain

^4^ Department of Rheumatology, Hospital Universitario Central de Asturias, Oviedo, Spain

**SUPPLEMENTARY TABLE 1: Genotype frequencies of the vitamin D-related polymorphisms studied.** The frequency of the genotypes of each polymorphism was calculated and differences between HC (n=87) and RA (n=194) were evaluated by χ2 tests.

| **SNP ID** | **HC**  **n (%)** | **RA**  **n (%)** | **p-value (**χ2 test**)** |
| --- | --- | --- | --- |
| VDR, rs2228750 |  |  |  |
| GG | 32 (36.3) | 71 (36.5) | 0.837 |
| AG | 40 (45.4) | 93 (47.9) |  |
| AA | 16 (18.1) | 30 (15.4) |  |
|  |  |  |  |
| CYP27A1, rs933994 |  |  |  |
| CC | 19 (21.5) | 49 (25.2) | 0.696 |
| CT | 50 (56.8) | 100 (51.5) |  |
| TT | 19 (21.5) | 45 (23.1) |  |
|  |  |  |  |
| CYP2R1, rs10741657 |  |  |  |
| GG | 33 (37.5) | 76 (39.1) | 0.908 |
| AG | 41 (46.5) | 85 (43.8) |  |
| AA | 14 (15.9) | 33 (17.0) |  |
|  |  |  |  |
| DHCR7, rs12785878 |  |  |  |
| TT | 37 (42.0) | 75 (38.6) | 0.820 |
| TG | 41 (46.5) | 93 (47.9) |  |
| GG | 10 (11.3) | 26 (13.4) |  |

**SUPPLEMENTARY TABLE 2: Effect of the different genetic polymorphisms analyzed on vitamin D serum levels in HC and RA.** HC and RA patients were stratified according to the genetic status for each of the polymorphisms analyzed: VDR-rs2228570, CYP27A1-rs933994, CYP2R1-rs10741657 and DHCR7-rs12785878. Vitamin D serum levels are indicated for each genotype and differences among genotypes were assessed by Kruskal Wallis tests (K-W test). Vitamin D serum levels are expressed as median (interquartile range).

| **SNP ID** | **HC** | **RA** |
| --- | --- | --- |
| **VDR, rs2228750** |  |  |
| GG | 27.80 (12.84) | 21.58 (16.51) |
| AG | 30.66 (11.90) | 25.06 (18.97) |
| AA | 32.32 (11.31) | 20.54 (12.97) |
| p-value (K-W test) | 0.603 | 0.195 |
|  |  |  |
| **CYP27A1, rs933994** |  |  |
| CC | 29.48 (10.19) | 26.89 (20.29) |
| CT | 29.69 (13.04) | 23.55 (15.03) |
| TT | 33.06 (20.14) | 19.04 (18.89) |
| p-value (K-W test) | 0.389 | 0.221 |
|  |  |  |
| **CYP2R1, rs10741657** |  |  |
| GG | 29.48 (11.59) | 19.70 (12.09) |
| AG | 30.21 (10.19) | 27.51 (12.09) |
| AA | 40.00 (20.48) | 26.16 (28.94) |
| p-value (K-W test) | 0.209 | 0.002 |
|  |  |  |
| **DHCR7, rs12785878** |  |  |
| TT | 29.44 (12.26) | 26.43 (16.95) |
| TG | 29.85 (12.80) | 19.86 (16.60) |
| GG | 24.59 (18.01) | 21.74 (17.24) |
| p-value (K-W test) | 0.454 | 0.038 |

**SUPPLEMENTARY TABLE 3: Characteristics of the study participants regarding genotyping status.** Variables were summarized as median (IQR), mean±SD or n(%). Statistical differences were assessed by Mann-Withney U or χ2 tests, as appropriate.

| **HC** |  | |  |
| --- | --- | --- | --- |
|  | **Genotyped**  **(n=88)** | **Not genotyped**  **(n=6)** | p-value |
| ***Demographical features*** |  |  |  |
| Age, years; median (range) | 51.83 (27.00 – 87.81) | 55.33 (19.00 – 87.00) | 0.010 |
| Gender, f/m | 53/16 | 121/21 | 0.132 |
| Sampling season, winter+spring / summer+autumn | 57/12 | 93/49 | 0.010 |
| ***Blood lipids, mean ± SD*** |  |  |  |
| Total-cholesterol, mg/dl | 203.72±31.69 | 208.63±37.18 | 0.523 |
| HDL-cholesterol, mg/dl | 50.30±17.01 | 62.71±16.67 | 0.003 |
| LDL-cholesterol, mg/dl | 120.81±27.28 | 123.45±34.65 | 0.795 |
| Total-/HDL-cholesterol ratio | 4.03±1.45 | 3.57±1.30 | 0.021 |
| Triglycerides, mg/dl | 103.00±56.23 | 99.75±65.54 | 0.494 |
| **RA** |  | |  |
|  | **Genotyped**  **(n=194)** | **Not genotyped**  **(n=17)** | p-value |
| ***Demographical features*** |  |  |  |
| Age, years; median (range) | 51.83 (27.00 – 87.81) | 55.33 (19.00 – 87.00) | 0.010 |
| Gender, f/m | 53/16 | 121/21 | 0.132 |
| Sampling season, winter+spring / summer+autumn | 57/12 | 93/49 | 0.010 |
| ***Blood lipids, mean ± SD*** |  |  |  |
| Total-cholesterol, mg/dl | 203.72±31.69 | 208.63±37.18 | 0.523 |
| HDL-cholesterol, mg/dl | 50.30±17.01 | 62.71±16.67 | 0.003 |
| LDL-cholesterol, mg/dl | 120.81±27.28 | 123.45±34.65 | 0.795 |
| Total-/HDL-cholesterol ratio | 4.03±1.45 | 3.57±1.30 | 0.021 |
| Triglycerides, mg/dl | 103.00±56.23 | 99.75±65.54 | 0.494 |
| ***Disease features*** |  |  |  |
| Disease duration, years | 2.16 (4.87) | 3.00 (5.31) | 0.079 |
| Age at diagnosis, years; median (range) | 48.73 (18.00 – 81.51) | 51.86 (18.00 – 87.81) | 0.068 |
| Disease activity (DAS28) | 3.79 (2.31) | 3.68 (2.13) | 0.608 |
| Tender Joint Count | 3.00 (7.00) | 3.00 (8.00) | 0.818 |
| Swollen Joint Count | 2.00 (5.00) | 1.00 (4.00) | 0.962 |
| Patient Global Assessment (0-100) | 40.00 (42.00) | 38.50 (41.00) | 0.591 |
| ESR, mm/h | 17.00 (23.00) | 18.00 (23.00) | 0.924 |
| CRP, mg/dl | 0.20 (0.54) | 0.20 (0.40) | 0.853 |
| HAQ (0-3) | 1.00 (1.13) | 0.75 (1.19) | 0.235 |
| Pain assessment (0-10) | 4.30 (4.00) | 4.00 (4.00) | 0.352 |
| RF (+), n(%) | 41 (59.4) | 77 (54.2) | 0.367 |
| ACPA (+), n(%) | 40 (57.9) | 40 (56.3) | 0.745 |
| Erosive disease (n=129), n(%) | 20 (28.9) | 30 (36.5) | 0.282 |
| ***Traditional CV risk factors, n(%)*** |  |  |  |
| Hypertension | 19 (27.5) | 46 (32.3) | 0.535 |
| Dyslipidemia | 20 (28.9) | 32 (22.5) | 0.396 |
| Diabetes | 9 (13.0) | 13 (9.1) | 0.376 |
| Smoking | 30 (43.4) | 45 (31.6) | 0.315 |
| BMI, mean ± SD | 28.30±5.17 | 26.53±4.40 | 0.089 |
| History of previous CVD | 17 (24.6) | 21 (14.7) | 0.080 |
| ***Treatments, n(%)*** |  |  |  |
| None | 20 (28.9) | 27 (19.0) | 0.101 |
| Glucocorticoids | 33 (47.8) | 71 (50.0) | 0.767 |
| Methotrexate | 43 (62.3) | 98 (69.0) | 0.365 |
| TNFα blockers | 14 (20.2) | 36 (25.3) | 0.402 |
| Tocilizumab | 5 (7.2) | 7 (4.9) | 0.751 |
| Vitamin D supplements | 9 (13.0) | (19.7) | 0.232 |

**SUPPLEMENTARY FIGURE 1: DHCR7 rs12785878 genetic variants influence DHCR7 serum levels in RA.** RA patients were stratified according to their genetic status of the DHCR7 rs12785878 polymorphism and the differences in the DHCR7 serum levels among genotypes were evaluated by a Kruskal Wallis test.


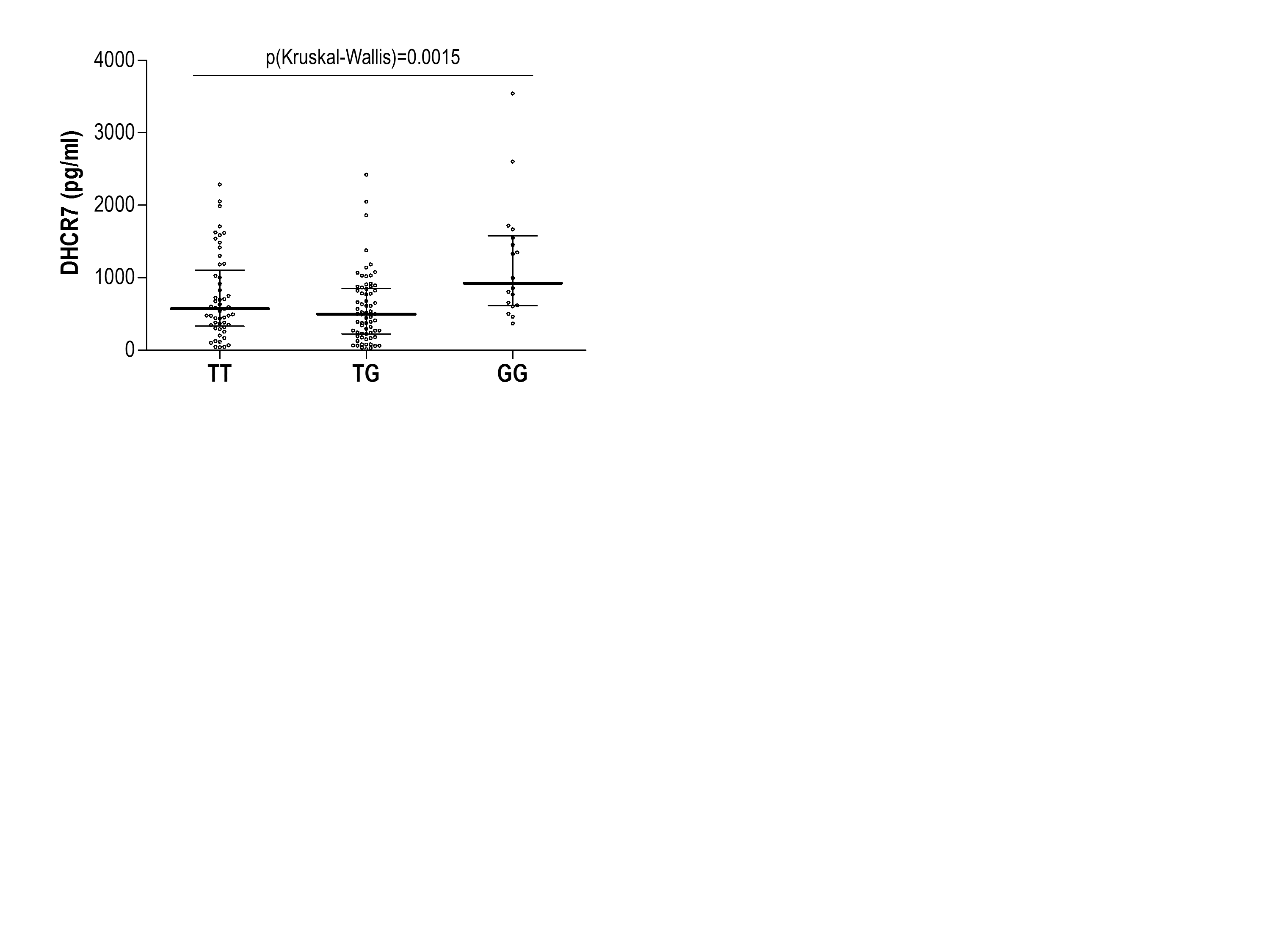

Supplement: Supplementary file 1 — Supplementary Materials [file 41598_2019_38756_MOESM1_ESM.docx]
